# Supplementary material for: Telework and Social Services in Spain during the COVID-19 Pandemic
Source: Int J Environ Res Public Health. 2021 Jan 15;18(2):725. doi: 10.3390/ijerph18020725 (PMC7830888; doi:10.3390/ijerph18020725)
Supplement: Supplementary file 1 [file ijerph-18-00725-s001.zip › Supplementary 2.pdf]

Supplementary 2

Dimension 2. Evaluation of the impact of covid-19 on professional development.

Descriptive statistics

	N	Minimum	Maximum	Media	Desv. típ.
The work we carry out from the social services has been greatly affected by the appearance of covid-19 and the state of alarm.	547	,00	4,00	3,4589	,96441
I think that in the service I work in we were sufficiently prepared for a situation like this.	557	,00	4,00	1,2496	1,18315
The response offered by the social services as a system has been adequate to the situation created.	552	,00	4,00	1,8750	1,19465
The population using social services is aware of the measures put in place during the state of alert.	544	,00	4,00	1,7132	1,22231
The population that uses social services appreciates the measures put in place in a positive way.	512	,00	4,00	1,9707	1,18583
Coordination between institutions to organise the response of social services as a system to covid-19 has been satisfactory.	545	,00	4,00	1,6477	1,25906
As professionals, we have had clear and concrete instructions on how to act in this situation.	547	,00	4,00	1,5137	1,28861
In my job, teleworking has allowed me to carry out my professional work normally.	530	,00	4,00	1,7585	1,37290
I have had sufficient means to telework during the development of the crisis.	518	,00	4,00	1,6737	1,41822
I have had the necessary training and instructions to be able to carry out my work telematically or not.	523	,00	4,00	1,7400	1,40240
The declaration of social service professionals as essential seems to me to be right.	422	,00	4,00	3,2085	1,20337
In general, it can be said that the social services system is overwhelmed by this situation.	453	,00	4,00	2,9448	1,14213
The available human resources are sufficient to develop our services during the state of alarm.	544	,00	4,00	1,4522	1,32323
My usual functions have been altered during the crisis period.	444	,00	4,00	2,9932	1,23619
The implementation of the new measures derived from the state of alarm has been done effectively and efficiently.	540	,00	4,00	1,7241	1,25422

Dimension 4. Evaluation of the impact on the personal and professional situation.

Descriptive statistics

	N	Minimum	Maximum	Media	Desv. típ.

I have often felt like crying these days.	556	,00	4,00	2,2230	1,47431
Throughout these days, discussions with social service colleagues have increased.	542	,00	4,00	1,2435	1,30961
I have often felt support and understanding from users of social services for the difficulty of the situation.	509	,00	4,00	2,4833	1,25735
During these weeks, I have worked many more hours beyond my working hours.	505	,00	4,00	2,3802	1,55648
It can be said that there have been times when I have felt overwhelmed by the situation.	530	,00	4,00	2,3755	1,43826
I have had the necessary protective equipment to do my job.	513	,00	4,00	2,5789	1,34850
My professional mobility has not been affected. I have been able to travel to my workplace without any problems.	501	,00	4,00	1,7126	1,50772
I have had the basic training to face my work during this time.	481	,00	4,00	2,3035	1,67063
At my workplace I have been properly accredited to do my job during the alarm period.	520	,00	4,00	1,7481	1,46375
In general, I have found support from my colleagues in solving the problems I have faced these days.	466	,00	4,00	2,7597	1,49773
I have often felt helpless these days.	473	,00	4,00	3,0803	1,23074
I'm teleworking at home and going to work one day.	477	,00	4,00	1,9937	1,74232
Despite teleworking, I know that at any time I can be called back to work.	437	,00	4,00	2,4485	1,66596

Dimension 5. Assessment of the adequacy of measures to address vulnerable populations.

Descriptive statistics

	N	Minimum	Maximum	Media	Desv. típ.
The strengthening and reorganisation of existing social resources by the municipal social services has been adequate.	507	,00	4,00	1,7988	1,23956
The municipality where I live has made an accurate diagnosis of the situation in which the most vulnerable populations find themselves during the health crisis.	480	,00	4,00	1,6771	1,23531
Collaboration with third sector entities is being fundamental to attend to these groups.	488	,00	4,00	2,6885	1,29654
The measures taken to ensure the confinement of vulnerable populations and segregated settlements have achieved their objective.	465	,00	4,00	2,0366	1,28350

Volunteers are doing a great job right now to help people in vulnerable situations.	460	,00	4,00	2,8283	1,26580
The measures taken for the socio-health monitoring of this population have allowed adequate attention to be given to it.	463	,00	4,00	2,0216	1,31737
The measures taken (financial or material assistance, processing of minimum income, extraordinary benefits...) to guarantee the income of these families are being managed rapidly.	473	,00	4,00	1,5962	1,36533
I believe that there are unmet vital needs.	493	,00	4,00	2,6146	1,40323
With regard to cash benefits, the creation of a fixed cash advance to ensure the immediate delivery of aid is working well.	380	,00	4,00	1,5553	1,38007
The systems implemented to replace the canteen grants and guarantee adequate food for the children are proving effective.	417	,00	4,00	2,2062	1,27123
The measures that have been developed to cater for early childhood (0-3 years) and pregnant women are appropriate.	342	,00	4,00	1,8129	1,23253
Programmes to monitor children's homework and social vulnerability are ensuring that their school performance is not delayed.	405	,00	4,00	1,5062	1,27353
In the service in which I develop my work, there is an effective coordination that allows to raise the knowledge of the professionals directly involved in the attention to the vulnerable population and higher levels of decision making.	520	,00	4,00	2,2154	1,41140
The paralysis of the social intervention projects (accompaniment) in relation to the social and labour inclusion, is causing a stop in the access to potential jobs of the vulnerable population.	460	,00	4,00	2,6696	1,40010
The paralysis of the administrative procedures for access to the Minimum Income of Insertion is aggravating the living conditions of the most vulnerable population.	454	,00	4,00	2,7996	1,37685
The suspension of Day Centres and Home Help Services that are not considered Minimum Services creates a problem of work-life balance for carers of dependent persons and an overload of their care tasks.	478	,00	4,00	2,8368	1,37152
Confinement has seriously affected people with mental health problems and mental illness and their families.	474	,00	4,00	3,0844	1,29287

Confinement is seriously affecting women victims of gender-based violence and their children.	459	,00	4,00	3,0610	1,33503
Most social service users do not have access to telematic procedures, and telephone assistance is sometimes not sufficient.	489	,00	4,00	2,8712	1,37519
The state of alarm and the health alert has made people vulnerable who, until now, were only in precarious conditions.	483	,00	4,00	3,0787	1,32875
In general, I believe that once the state of alarm and health alert has passed, the living conditions of the vulnerable population will have worsened.	487	,00	4,00	3,1191	1,28067
