# Supplementary material for: Telework and Social Services in Spain during the COVID-19 Pandemic
Source: Int J Environ Res Public Health. 2021 Jan 15;18(2):725. doi: 10.3390/ijerph18020725 (PMC7830888; doi:10.3390/ijerph18020725)
Supplement: Supplementary file 1 [file ijerph-18-00725-s001.zip › Supplementary 1.pdf]

Supplementary 1

Questionnaire Social Services and COVID-19

The main objective pursued by this instrument is to assess the impact of the coronavirus crisis and the state of alarm decree on the development of social services on the one hand, and on the other, on the lives of people, cohabitation units and vulnerable groups who come to social services.

The target audience is made up of social service professionals as a whole, both in terms of primary care services in all their forms, services linked to dependency and specialised services, both public and private.

It has three distinct parts: the first is a set of questions related to the identification data of the person answering the survey. The second, centred on covid-19, aims to assess the impact of this on the provision of services, the knowledge of the measures implemented and the evaluation of these. The third focuses on assessing how this situation affects the most vulnerable groups.

1. SOCIODEMOGRAPHIC DATA

Please tell us:

1. **Sex or gender**
   Man:
   Woman:
   Other: Which one?

2. **Age:**
   [ ] (complete)

3. **Marital status:**
   1. Single
   2. Divorced / separate
   3. Married / domestic partnership / living together as a couple
   4. Widower

4. **Do you have children?**

   1. Yes.
   2. Not.

5. **How many children live with you?**
   [ ] (complete)

6. **Level of studies (indicate the highest):**
   1. No studies.
   2. Primary studies (EGB, ESO).
   3. Secondary studies (FP, BUP, COU, BACCALAUREATE).
   4. University studies (Diploma, Bachelor, Degree).
   5. Master's degree.
   6. Doctorate.

7. In case of university studies, please indicate your degree?

0. Not applicable.
1. Social Work.
2. Psychology.
3. Social Education.
4. Physiotherapy.
5. Occupational therapy.
6. Law.
7. Other. Which one?

8. Labour situation:

1. Full-time worker
2. Part-time worker
3. Volunteering

9. Time in years you have worked in your current entity/organization/service
[ ] (complete)

10. tipo de organización en la que trabaja:

1. Public, Town Hall.

2. Public, Provincial Council.

3. Public, service dependent on the Autonomous Community

4. Public, a service dependent on the State Administration.

5. Third sector (association, foundation...)

6. Social services company.

7. Other, which one?

11. Indique colectivo principal con el que desarrolla su actividad profesional (exclusión social, personas con discapacidades, personas migrantes, violencia de género, menores, mayores, etc. o servicio generalista –atiende a todos los colectivos).

[ ] (complete)

12. In which location do you carry out your activity?

[ ] (completar)

13. What position do you hold in your workplace?

1. Management functions, coordination, team responsibility.
2. Programme manager.
3. Technician.
4. Volunteer work.
5. Other. Which one?

2. ASSESSMENT OF THE IMPACT OF COVID-19 AND THE STATE OF ALARM ON THE DEVELOPMENT OF SOCIAL SERVICES

State your degree of agreement or disagreement with the following statements. Remember that 1 equals very much in disagreement and 5 equals very much in agreement. You may also not answer or indicate that you do not know the answer.

1. The work we carry out from the social services has been greatly affected by the appearance of covid-19 and the state of alarm.
2. I think that in the service where I work we were sufficiently prepared for a situation like this.
3. The response offered by the social services as a system has been adequate to the situation created.
4. The population using social services is aware of the measures put in place during the state of alarm.
5. The population using social services has a positive view of the measures implemented.
6. Coordination between institutions to organise the response of social services as a system to the covid-19 has been satisfactory.
7. As professionals, we have had clear and concrete instructions on how to act in this situation.
8. In my job, teleworking has allowed me to carry out my professional work normally.
9. I have had sufficient means to telework during the development of the crisis.
10. I have had the necessary training and instructions to be able to carry out my work telematically or not.
11. The declaration of social service professionals as essential seems to me to be correct.
12. In general, it can be said that the social services system is overwhelmed by this situation.
13. The available human resources are sufficient to develop our services during the state of alarm.
14. My usual functions have been altered during the crisis period.
15. The implementation of the new measures derived from the state of alert has been done effectively and efficiently.

Please tell us about any experience and/or professional situation that has caught your attention or impacted you during this time. Or point out one or more alternative proposals to the measures developed. Please also assess your perception of the future of social services.

3. **INDICATE WHETHER OR NOT YOU ARE AWARE OF THE PROTECTION AND INFORMATION ACTIONS CARRIED OUT IN THE WORK CENTRES**

Answer with a yes, a no or I don't know.

In my workplace:

1. Messages or circulars have been prepared and posters have been placed at the entrance, indicating to visitors that they should not go to these centres unless it is essential and in no case if they present any respiratory symptoms or fever.
2. Information posters on hand hygiene and respiratory hygiene have been placed at the entrance to the centres, corridors and common areas.
3. Training activities have been carried out for the health education of users and workers on hand and respiratory hygiene.
4. It has been guaranteed that all toilets have soap and paper towels for hand hygiene.
5. Hydro-alcoholic solution dispensers for hand hygiene, disposable tissues for respiratory hygiene and waste containers with pedal-operated covers have been provided.
6. Measures have been taken to minimise interaction with users and other professionals
7. Intensified cleaning programmes have been implemented in the centre, with special attention to areas where the greatest number of people can transit and to surfaces of frequent contact such as handrails and rails, buttons, door knobs, tables, etc.
8. Workers and users of the centre have been informed of the actions being taken to protect them.

4. **ASSESS YOUR PERSONAL PROFESSIONAL SITUATION DURING THE ALARM STATE**

Remember that 1 equals very much in disagreement and 5 equals very much in agreement. You may also not answer or indicate that you do not know the answer.

1. I have often felt like crying these days.
2. Throughout these days, discussions with colleagues from social services have increased.
3. I have often felt support and understanding from social service users for the difficulty of the situation.
4. During these weeks, I have worked many more hours beyond my working hours.
5. It can be said that there have been times when I have felt overwhelmed by the situation.
6. I have had the necessary protective equipment to do my job.
7. My professional mobility has not been affected. I have been able to travel to my workplace without problems.
8. I have had basic training to cope with my work during this time.

9. I have been properly accredited at my workplace to do my job during the alarm period.

10. In general, I have found support from my colleagues in solving the problems I have faced these days.

11. I have often felt powerless these days.

12. I am teleworking at home and go to the workplace one day.

13. Despite teleworking, I know that at any time I can be called to work.

5. ASSESSMENT OF THE ACTIONS IMPLEMENTED FOR THE PROTECTION OF VULNERABLE GROUPS

How do you assess the set of actions developed in your environment to protect the most vulnerable groups during the cvod-19 crisis and the state of alarm, taking into account that 1 is a very negative assessment and 5 a very positive one. You may also not answer or indicate that you do not know the answer.

1. The strengthening and reorganisation of existing social resources by the municipal social services has been adequate.

2. The municipality where I live has made an accurate diagnosis of the situation of the most vulnerable populations during the health crisis.

3. Collaboration with third sector organisations is essential to attend to these groups.

4. The measures taken to guarantee the confinement of vulnerable populations and segregated settlements have achieved their objective.

5. The voluntary sector is doing a great deal of work at the moment to attend to people in vulnerable situations.

6. The measures taken for the social and health monitoring of this population have allowed adequate attention to be given to them.

7. The measures taken (economic or material assistance, processing of minimum income, extraordinary benefits...) to guarantee the income of these families are being managed quickly.

8. I consider that there are unmet vital needs.

9. As far as cash benefits are concerned, the creation of a fixed cash advance to guarantee the immediate delivery of aid is working well.

10. The systems implemented to replace the canteen grants and to guarantee adequate food for the children are proving effective.

11. The measures that have been developed to attend to early childhood (0-3 years) and pregnant women are adequate,

12. Programmes to monitor children's homework and social vulnerability are ensuring that their school performance is not delayed

13. In the service in which I carry out my work, there is effective coordination that allows me to increase the knowledge of the professionals directly involved in the care of vulnerable populations and higher levels of decision making.

14. The paralysis of social intervention projects (accompaniment) in relation to social and labour inclusion is causing a halt in access to potential jobs for the vulnerable population.

15. The paralysis of administrative procedures for access to the minimum income is aggravating the living conditions of the most vulnerable population.

16. The suspension of Day Care Centres and Home Help Services that are not considered Minimum Services creates a problem of work-life balance for carers of dependent persons and an overload of their care duties (work responsibilities, care of children and elderly people).

17. Confinement has seriously affected people with mental health problems and mental illness and their families.

18. Confinement is seriously affecting women victims of gender-based violence and their children.

19. Most social service users do not have access to telematic procedures, and telephone assistance is sometimes not sufficient.

20. The state of alarm and the health alert has made the population vulnerable, which until now was only in precarious conditions.

21. In general, I believe that once the state of alarm and health alert has passed, the living conditions of the vulnerable population will have worsened.

I understand that it can take a long time, but I would ask the 3 questions separately and in reverse order to prioritize the most important questions. I insist that this part is very important, because certain very vulnerable groups could be left out of the measures... I would change the question by leaving it like this

Reflections on the future of the vulnerable population What cases or profiles will be most affected and why?

What alternatives or measures do you consider have not been taken into account in the measures implemented by the competent governments and which are necessary for the care of vulnerable groups?

Tell us about an experience or situation that has caught your attention during this time.

Thank you very much for your collaboration and for your work during these days.
